# Stroke lesion in cortical neural circuits and post-stroke incidence of major depressive episode: A 4-month prospective study

**DOI:** 10.3109/15622975.2011.562242

**Published:** 2011-04-12

**Authors:** Luisa Terroni, Edson Amaro, Dan V Iosifescu, Gisela Tinone, João Ricardo Sato, Claudia Costa Leite, Matildes F M Sobreiro, Mara Cristina Souza Lucia, Milberto Scaff, Renério Fráguas

**Affiliations:** 1 Liaison Psychiatry Group, Department and Institute of Psychiatry, Clinical Hospital, Medical School, University of São Paulo, São Paulo, Brazil; 2 Department of Radiology, Clinical Hospital, Medical School, University of São Paulo, São Paulo, Brazil; 3 Mood and Anxiety Disorders Program, Mount Sinai School of Medicine, New York, NY, USA; 4 Department of Neurology, Clinical Hospital, Medical School, University of São Paulo, São Paulo, Brazil; 5 Center of Mathematics, Computation and Cognition, Federal University of ABC and Department of Radiology, Clinical Hospital, Medical School, University of São Paulo, São Paulo, Brazil; 6 Liaison Psychiatry Group, Department and Institute of Psychiatry, Clinical Hospital, Medical School, University of São Paulo, São Paulo, Brazil; 7 Department of Neurology, Division of Psychology, Clinical Hospital, Medical School, University of São Paulo, Brazil; 8 Liaison Psychiatry Group, Laboratory of Psychiatric Neuroimaging (LIM-21), Department and Institute of Psychiatry, Clinical Hospital, Medical School, University of São Paulo, São Paulo, Brazil

**Keywords:** Cingulate gyrus, depression disorder, magnetic resonance imaging, prefrontal cortex, stroke

## Abstract

**Objective:**

Little is known about the relevance of lesion in neural circuits reported to be associated with major depressive disorder. We investigated the association between lesion stroke size in the limbic-cortical-striatal-pallidal-thalamic (LCSPT) circuit and incidence of major depressive episode (MDE).

**Methods:**

We enrolled 68 patients with first-ever ischemic stroke and no history of major depressive disorder. Neurological and psychiatric examinations were performed at three time-points. We diagnosed major depressive episode, following DSM-IV criteria. Lesion location and volume were determined with magnetic resonance imaging, using a semi-automated method based on the Brodmann Cytoarchitectonic Atlas.

**Results:**

Twenty-one patients (31%) experienced major depressive episode. Larger lesions in the left cortical regions of the LCSPT circuit (3,760 vs. 660 mm^3^; *P* = 0.004) were associated with higher incidence of MDE. Secondary analyses revealed that major depressive episode was associated with larger lesions in areas of the medial prefrontal cortex including the ventral (BA24) and dorsal anterior cingulate cortex (BA32) and subgenual cortex (BA25); and also the subiculum (BA28/36) and amygdala (BA34).

**Conclusions:**

Our findings indicate that depression due to stroke is aetiologically related to the disruption of the left LCSPT circuit and support the relevance of the medial prefrontal cortex dysfunction in the pafhophysiology of depression.

## Introduction

The reported prevalence of major depression within 3 months after stroke ranges from 22 to 31% (Robinson et al. 1984b; Astrom et al. 1993; Terroni et al. 2003; Spalletta et al. 2005). Efforts to identify biological and psychosocial mechanisms have provided evidence that the aetiology of post-stroke depression is multifactorial (Robinson et al. 1984a; Astrom et al. 1993; Andersen et al. 1995; Vataja et al. 2001). However, research focusing on stroke location has been a fruitful strategy in understanding the pathophysiology of post-stroke depression (Robinson et al. 1984a; Mayberg et al. 1988; Astrom et al. 1993; Vataja et al. 2001; Vataja et al. 2004). From a clinical perspective, knowing which patients are at increased risk of developing post-stroke depression may ameliorate the prevention, detection, and early treatment of depression, consequently reducing its the negative impact on the recovery of stroke patients (Ramasubbu and Kennedy 1994).

Although there is no consensus about the relationship between lesion location and post-stroke depression (Singh et al. 1998; Carson et al. 2000; Bhogal et al. 2004; Hackett and Anderson 2005), some studies using computed tomography, magnetic resonance imaging (MRI) (Robinson et al. 1984a; Astrom et al. 1993; Vataja et al. 2001, 2004) and PET imaging of cortical S2 serotonin receptors (Mayberg et al. 1988) have suggested that post-stroke depression is associated with the proximity of the lesion to the frontal lobe and with left hemisphere stroke. In addition, studies in major depressive disorder with PET (Baxter et al. 1989; Drevets et al. 1992), and with catecholamine depletion (Hasler et al. 2008), have found abnormal prefrontal function, more commonly in the left than in the right hemisphere.

MRI studies (Vataja et al. 2001,^2004^) have reported a high prevalence of post-stroke depression in lesions affecting some structures of the prefronto-subcortical circuit, particularly in the left hemisphere, a circuit that has been reported to be involved in various neu-ropsychiatry syndromes, including depression. Recent studies have highlighted the specific relevance of the limbic-cortical-striatal-pallidal-thalamic (LCSPT) circuit in the pathophysiology of major depressive disorder (Drevets et al. 2008; Hasler et al. 2008). Based on evidence from animal studies, Drevets et al. (2008) proposed that in addition to the LCPSPT circuit, two other circuits are essential for emotional regulation. The first, the orbital prefrontal network, is involved in a system of reward, aversion, and relative value. The second, the medial prefrontal network, has connections with limbic and visceral control structures that are involved in introspective functions such as mood and emotion, and visceral reactions to emotional stimuli such as autonomic regulation and neuroendocrine responses. The prefrontal cortex, which includes areas belonging to all these circuits, has been implicated in response to treatment for major depressive disorder (Mayberg et al. 1997; Brody et al. 1999; Pizzagalli et al. 2001).

To our knowledge, there have been no studies investigating the relationship between stroke lesions in LCSPT circuit and the development of post-stroke depression. Therefore, the primary aim of the present study was to investigate the association between lesion volume in the left LCSPT circuit and the incidence of major depressive episode in the first four months after stroke. We focused on the left hemisphere as the left lateralization of stroke has been repeatedly associated with post-stroke depression (Robinson et al. 1984a; Astrom et al. 1993; Vataja et al. 2001, 2004; Bhogal et al. 2004). As secondary hypothesis, we investigated the association between the incidence of major depressive episode and lesions involving cortical regions of the orbital and medial prefrontal networks. When cortical circuits were found to be statistically associated with post-stroke depression, we also investigated the relationship between lesions in specific brain areas incorporated in those circuits and incidence of major depressive episodes.

## Methods

### Patients

**Figure 1:**
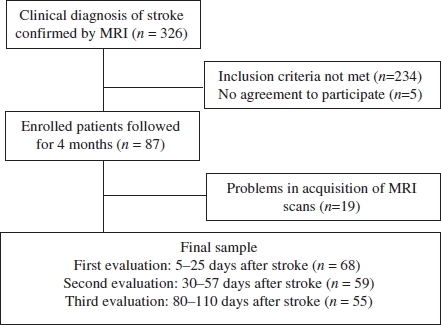
Patient flow.

We screened 326 male and female patients, 18 years of age or older, consecutively admitted to the Neurology Unit of a University Hospital with a diagnosis of ischemic stroke between August of 2002 and May of 2008. The diagnosis of stroke was made by a neurologist in accordance with the World Health Organization criteria (WHO 1989) and confirmed by MRI. A psychiatrist administered the modules for mood episodes, psychotic symptoms and substance use disorders of the Structured Clinical Interview (SCID) for Diagnostic and Statistical Manual of Mental Disorders fourth edition (DSM-IV) to investigate past and current psychiatric disorders (American Psychiatric Association 1994; First 1995). This interview was performed with the patient and a family member/caregiver present when possible. Patients with previous history of stroke or other central nervous system diseases (i.e. amyotrophic lateral sclerosis, subarachnoid haemorrhage, Binswanger's disease, brain tumours, or multiple sclerosis) were excluded from the study, as were those with infratentorial stroke, a severe clinical condition that impeded the interview, Cushing's syndrome, alcohol or drug dependence in the last 12 months, previous history of major depressive episode or bipolar disorder, current major depressive episode or bipolar disorder with pre-stroke onset, psychotic disorder, dementia, or aphasia that impeded the interview. On the basis of these criteria, we excluded 234 patients (Figure 1): history stroke, infratentorial stroke, greater than 2-week interval between stroke occurrence and screening interview, or haemorrhagic transformation of stroke (*N* = 89); drug/alcohol dependence, psychoses, delirium, history of major depressive episode, current major depressive episode with pre-stroke onset, dysthymia, or bipolar disorder (*N* = 54); aphasia that impeded the interview (*N* = 37); neurological diseases or severe clinical condition that impeded the interview (*N* = 22); and other reasons (*N* = 32). Of the remaining 92 patients, five declined to participate in the study. Of those 87 patients, 19 were later excluded due to problems during the MRI acquisition: image was inappropriate for use in the present study (*N* =15); claustrophobia (*N* = 3); and haemorrhagic transformation of stroke (*N* = 1). Therefore, we enrolled a final sample of 68 patients. Nine patients dropped out after the first time-point examination and four patients dropped out after the second time-point examination. The institutional review board of the Clinics Hospital approved the study protocol and, written informed consent was obtained from all participating patients.

### Clinical assessments

The enrolled patients were evaluated at three different time-points: between post-stroke days 5 and 25 (mean ± SD; 12.0 ± 4.5; range, 5–23 days); between post-stroke days 30 and 59 (37.0 ± 6.0; 30–57 days); and, between post-stroke days 80 and 110 (91.6 ± 5.4; 83–108 days). In all three evaluations, the diagnosis of major depressive episode was made by an experienced psychiatrist (LT), blinded for imaging data, using the SCID for DSM-IV, Axis I disorders (American Psychiatric Association 1994; First 1995). In the first evaluation in the Neurology Unit, as we described in a previous work (Terroni et al. 2009), the diagnosis of major depressive episode was made considering a period of 1 week, as have been done in others studies (Robinson et al. 1984a; Astrom et al. 1993; Caeiro et al. 2006). The 31-item Hamilton Rating Scale for Depression (HAM-D-31) (Williams 1988; Jamerson et al. 2003) was used at every visit to assess the severity of depressive symptoms. At all three time-points, a neurologist (GT), certified by the National Institutes of Health to administer the National Institutes of Health Stroke Scale (NIHSS) (Brott et al. 1989), blinded for imaging data and psychiatric diagnoses, evaluated the stroke severity using the NIHSS and the impairment of activities of daily living using the Barthel Index (Herndon 1997). Cognitive performance was assessed at the first and third time-points with the Mini-Mental State Examination (MMSE) (Folstein et al. 1975) administered by a neuropsychologist (MS) blinded for imaging data and psychiatric diagnoses.

### MRI methods

The MRIs were acquired in general within two weeks after stroke (9.34 ± 6.87; range 1–43 days). All images were acquired using a 1.5-Tesla system (GE-Horizon LX). The imaging protocol included axial spoiled gradient recalled acquisition in steady state (SPGR, TR = 27 ms; flip angle = 45°; voxel size = 0.94 × 0.94 × 1.5 mm), axial fluid attenuated inversion recovery (FLAIR, TR = 133 ms; TE = 8400 ms; TI = 2100 ms; voxel size = 0.94 × 0.94 × 5 mm), axial diffusion-weighted image (TR = 8000 ms; *b* value = 1000 s/mm^2^; voxel size = 1.8 × 1.8 × 5 mm), T2-weighted fast spin echo (TR = 4500 ms; TE = 100–120 ms; voxel size = 0.94 × 0.94 × 5 mm). All images were acquired in the bicommissural plane.

Lesion location and volume quantification were determined using a semi-automated method. Initially, SPGR and axial FLAIR acquisitions were both normalized to the Montreal Neurological Institute template (Evans et al. 1993) using linear transformation with 12 degrees of freedom and 15 nonlinear interactions implemented in Statistical Parametric Mapping (SPM5,Wellcome Trust for Neuroimaging, London, http://www.fil.ion.ucl.ac.uk/spm/) (Friston et al. 1996), and based on coordinates referenced in the Talairach and Tournoux Atlas (Talairach and Tornoux 1988). During this process, all images were sampled to 2.3 × 2.3 × 2.6 mm. Lesion delineation was performed by a trained psychiatrist (LT) using a mouse device to trace the ischemic lesion and analyzing all slices of each FLAIR image using MRIcro Software (http://www.sph.sc.edu/comd/rorden/mricro.html) (Rorden and Brett 2000). All lesions delineations of each patient were reviewed by a neuroradiologist (EAJ), blinded for clinical data and psychiatric diagnoses.

RF had elaborated the main hypothesis of the study related to the lesions in the LCSPT and post-stroke depression but did not disclose it to the raters of MRI lesions (LT, EA) while lesion delineation was taking place, in order to preserve the blinding. Both raters delineating MRI lesions (LT, EAJ) were only aware they would study size and location of the lesions using the Brodmann Map in relation to post stroke depression. Both raters (LT, EA) were also blind to the clinical outcome status (post stroke depression episodes) of the patients while delineating lesions on MRIs. After finishing the lesion delineation process, RF (who had not participated in lesion delineation) revealed the specific hypothesis to be tested. Diffusion-weighted images were also analyzed in order to distinguish between other possible differential diagnoses. The regions of interest were then analyzed automatically using the Brodmann Cytoarchitectonic Atlas registered to the same space (Van Essen and Drury 1997; Van Essen et al. 1998), in order to count the number of voxels within each Brodmann area (BA). The total lesion volume was obtained by multiplying the number of voxels by voxel size in normalized images. Lesions in the white matter substance were classified with the Fazekas scale, which provides an assessment of severity of the white-matter hyperintensities in the periventricular area (PWMH) and in the deep white-matter (DWMH) (Fazekas et al. 1987; Iosifescu et al. 2006). This process was made by a neuroradiologist (EA) blinded to the clinical status of the patients.

### Neural circuit: definitions

Our main hypothesis was that post-stroke major depressive episode was associated with lesion volume in the LCSPT circuit (Drevets et al. 2008) in the left hemisphere.

The LCSPT circuit is composed of connections between the orbital prefrontal cortex and medial prefrontal cortex (Ongur et al. 2003). We selected the BAs included in each of these circuits according to their neuroanatomical definitions (Brodmann 1909; Ongur and Price 2000; Ongur et al. 2003; Drevets et al. 2008).The BAs included in the LCSPT circuit are: BAH, BA12, BA13 and BA47 (orbital prefrontal cortex); BA9, BA10, BAH, BA13, BAH, BA24, BA25, BA32 and BA47 (medial prefrontal cortex); BA34 (amygdala); and BA28/BA36 (corresponding to the subiculum). Subcortical structures of the LCSPT circuit were not included because the Brodmann map comprises primarily cortical structures.

As secondary hypotheses, we investigated the association between post-stroke major depressive episode and two networks (Drevets et al. 2008).The orbital prefrontal network includes the orbital prefrontal cortex together with the following areas: BA20 (inferior temporal cortex); BA13/BA14 (insula); BA44/ BA45 (frontal operculum); BA27/BA34 (olfactory cortex); and BA43 (taste cortex). The second, the medial prefrontal network including the medial prefrontal cortex, together with the following areas: BA23/BA31 (mid/posterior cingulate cortex); BA22/ BA38 (anterior superior temporal gyrus/sulcus); BA28/BA34 (entorhinal cortex); and BA35/BA36 (posterior parahippocampal cortex).

### Statistical analysis

The lesion volume for a given circuit was obtained by determining the voxel-based lesion morphometry in that circuit. To test our main hypothesis we investigated the association of lesion volume in the left LCSPT with the incidence of post-stroke major depressive episode. To test our secondary hypotheses we investigated whether major depressive episode incidence was associated with the orbital prefrontal network and medial prefrontal network in the left hemisphere and with the three target circuits in the right hemisphere. When we found a significant association for a given circuit, we performed complementary analyses to investigate whether a lesion volume in a specific BA in that circuit was associated with the incidence of major depressive episode. Two patients with bilateral stroke were excluded from the analyses. Lesion volume is expressed as mean and standard deviation of voxels in mm^3^ (FLAIR voxel size 2.3 × 2.3 × 2.6 mm).

Statistical analyses were performed using the Statistical Package for the Social Sciences, version 14 (Chicago: I1, SPSS inc., 2005). The Chi-square test or Fisher's exact test were used for categorical data, and the *t*-test was used for continuous variables, and the Mann-Whitney *U*/-test was used when data did not have normal distribution according to the Kolmogorov-Smirnov test. Results are presented as frequencies, values of the rank mean and rank sum in the Mann-Whitney *U*/-test, and also mean ± standard deviation. All statistical tests were based on two-tailed significance.

To account for multiple comparisons and to minimize type I errors we followed Hochberg's recommendations (Hochberg 1988). Thus, we tested our main hypothesis related to left sided LCSPT circuit at α = 0.05, our hypothesis related to left sided orbital prefrontal network at α = 0.025 and the hypothesis related to left sided medial prefrontal network at α = 0.0166. For the right sided circuits we tested at the following *P* values: right LCSPT circuit at α = 0.0125, right orbital prefrontal network at α = 0.01 and right medial prefrontal network at α = 0.0083. The order of these comparisons was pre-determined by our hypotheses. For circuits where overall *P* values were statistically significant after multiplicity adjustments we investigated associations with individual BAs at *P* = 0.05 level.

## Results

### Demographic and clinical data

Twenty-one patients (31%) were diagnosed with a new-onset major depressive episode, including seven at the time of the first evaluation, five at the second and, nine at the third evaluation. The depressed patients were comparable to those without major depressive episode in terms of sociodemographic, clinical, neurological, lesions in white matter, and cognitive aspects, with the exception of an increased rate of Diabetes Mellitus among depressed patients (Table I). Thirty-nine patients (57.4%) had suffered a left hemisphere stroke, 27 (39.7%) had suffered a right hemisphere stroke and 2 (2.9%) had suffered a bilateral stroke.

**Table I:** Demographic and clinical characteristics of patients with and without major depressive episode after stroke.

	Major depressive episode		
		
	Yes *N* (%)	No *N* (%)	*P* value
Patients	21 (31)	47 (69)	
Sex			
Female	10 (48)	22 (47)	
Male	11 (52)	25 (53)	0.951
Married			
yes	15(71)	29 (62)	
no	6(29)	18 (38)	0.606
Level of educational			
≤8 years of schooling	16 (76)	32 (68)	
≥9 years of schooling	5(24)	15 (32)	0.435
Employed			
yes	11 (52)	32 (68)	
no	10 (48)	15 (32)	0.215
Left hemisphere lesion			
yes	13 (62)	28 (59.6)	
no	8(38)	19 (40.4)	0.856
Right hemisphere lesion			
yes	9 (42.9)	20 (42.6)	
no	12 (57.1)	27 (57.5)	0.981
Handeness^b^			
Right-handed	21 (100)	44 (93.6)	
Left-handed	0(0)	3 (6.4)	0.547
HTN^c^			
Yes	12 (57.1)	22 (50)	
No	9 (42.9)	22 (50)	0.590
DM^b^,^c^			
Yes	6 (28,6)	3 (6.8)	
No	15 (71.4)	41 (93.2)	0.048
CHD^b^,^c^			
Yes	1 (5)	1 (2.2)	
No	19 (95)	44 (97.8)	0.524
Dysphasia^b^,^d^			
Yes	1 (4.8)	8(19)	
No	20 (95.2)	34 (81)	0.251
White-matter hyperintensity score^e^			
PWMH			0.182
PWMH = 0	2 (9.5)	2 (4.3)	
PWMH = 1	13 (61.9)	39 (83)	
PWMH = 2	5 (23.8)	6 (12.8)	
PWMH = 3	1 (4.8)	0(0)	
DWMH			0.486
DWMH = 0	6 (28.6)	12 (25.5)	
DWMH = 1	10 (47.6)	23 (48.9)	
DWMH = 2	3 (14.3)	11 (23.4)	
DWMH = 3	2 (9.5)	1 (2.1)	
	Mean (SD)	Mean (SD)	
Age, years	53.8 (15.8)	49.8 (12.8)	0.280
NIHSS^a^			
First time-point	3.7 (3.0)	2.7 (2.7)	0.134
Second time-point	2.8 (2.4)	2.4 (2.7)	0.379
Third time-point	2 (1.8)	1.9 (1.9)	0.825
Barthel Index^a^			
First time-point	86 (24.8)	92.6 (19.1)	0.123
Second time-point	91.3 (17.4)	93.3 (17.1)	0.366
Third time-point	97.4 (5.4)	96.4 (9.5)	0.901
MMSE			
First time-point	23.5 (4.2)	23.7 (4.5)	0.933
Third time-point	24.5 (4.3)	24.4 (3.7)	0.900
HAM-D			
First time-point	23 (2.9)	5.7 (3.9)	<0.001
Second time-point	22.2 (6.8)	5.7 (4.6)	<0.001
Third time-point	20.2 (4)	5.1 (5)	<0.001

NIHSS, National Institutes of Health Stroke Scale; MMSE, Mini-Mental State Examination; HAM-D, Hamilton Rating Scale for Depression, 31-item version; HTN, hypertension; DM, diabetes mellitus; CHD, coronary heart disease; PWMH, periventricular hyperintensity; DWMH, deep white-matter hyperintensity.

a Mann–Whitney test;

b Fischer's exact test;

c *N* = 65, three cases with no data;

d *N* = 63, five cases with no data;

e Fazekas Score.

There were no significant differences between the patients included in the final study (*N* = 68) and those who had been excluded because of problems in the MRI acquisitions (*N* = 19) in terms of sociodemographic, neurological, and cognitive measures, except for the fact that NIHSS scores were lower in the studied patients at the first time-point, but not at the second and third time-points.

### Analysis of lesion location and volume in neural circuit

Patients with first major depressive episode after stroke had larger lesion volume in the left LCSPT circuit than non-depressed patients (respectively 3,760 vs. 660 mm^3^; *P* = 0.004; Table II). The incidence of major depressive episode was also associated with lesion volume in the left orbital prefrontal network (*P* = 0.037), our secondary hypothesis, but this comparison did not reach statistical significance after Hochberg's multiplicity adjustments (*P* = 0.025). Complementary analyses revealed that major depressive episode incidence was associated with specific areas of the left LCSPT circuit (Table III) including the ventral anterior cingulate cortex (BA24), subgenual cortex (BA25), subiculum (BA28/BA36), amygdala (BA34), and dorsal anterior cingulate cortex (BA32).

**Table II:** Association between lesion volume in neural circuits and four-month incidence of major depressive episode after ischemic stroke.

	Major depressive episode^a^		
		
	Yes^b^	No^c^	*P* value^d^
*Left hemisphere* (*N* = 39)^e^	*N* = 12 (30.8%)	*N* = 27 (69.2%)	
LCSPT	3,760 (5,840)	660 (3,080)	0.004
	26.21 (314.5)	17.24 (465.5)	
OPFN	4,900 (10,080)	1,680 (6,230)	0.037
	25.25 (303)	17.67 (477)	
MPFN	4,380 (6,350)	860 (3,170)	0.065
	24.58 (295)	17.96 (485)	
*Right hemisphere* (*N* = 27)^e^	*N* = 8 (29.6%)	*N* = 19 (70.4%)	
LCSPT	1,270 (1,610)	6,170 (15,650)	0.697
	14.88 (119)	13.63 (259)	
OPFN	3,900 (4,120)	6,300 (10,830)	0.667
	15.00 (120)	13.58 (258)	
MPFN	3,450 (2,720)	7,010 (15,840)	0.435
	15.81 (126.5)	13.24 (251.5)	

LCSPT, limbic-cortical-striatal-pallidal-thalamic circuit; OPFN, orbital prefrontal network; MPFN, medial prefrontal network.

a Measurement of size is in mm^3^ (mean ± SD) in the upper line and value of mean rank (rank sum) in the below line.

b One patient with bilateral stroke was excluded of this analysis.

c One patient with bilateral stroke was excluded of this analysis.

d Mann–Whitney test with *P* values of the Hochberg's recommendations: left sided LCSPT circuit at α = 0.05, left sided OPFN at α = 0.025, left sided MPFN at α = 0.01667, right LCSPT circuit at α = 0.0125, right OPFN at α = 0.01 and right MPFN at α = 0.0083.

e Final sample in the group of patients.

**Table III:** Complementary analysis of the association of lesion volume in areas of the LCSPT in the left hemisphere and incidence of major depressive episode.

	Major depressive episode^a^		
		
	Yes (*n* = 12)^c^	No (*n* = 27)^c^	*P* value^b^
Amygdala (BA34)	87 (160)	4 (21)	0.010
	24.08 (289)	18.19 (491)	
Ventral anterior	690 (1,990)	0(0)	0.032
cingulate cortex (BA24)	22.25 (267)	19.00 (513)	
Subgenual cortex	280 (710)	4 (20)	0.038
(BA25)	23.00 (276)	18.67 (504)	
Hippocampal	39 (130)	0 (0)	0.032
subiculum (BA28)	22.25 (267)	19.00 (513)	
Dorsal anterior	740 (2,190)	5 (24)	
cingulate cortex (BA32)	22.92 (275)	18.70 (505)	0.043
Hippocampal	40 (120)	0 (0)	0.032
subiculum (BA36)	22.25 (267)	19.00 (513)	

BA, Brodmann area.

a Measurement of size is in mm^3^ (mean ± SD) in the upper line and value of mean rank (rank sum) in the below line.

b Mann–Whitney test without multiple comparison adjustment.

c Final sample in the group of patients.

Due to the increased rate of diabetes mellitus among depressed patients and its potential confounding effect, we investigate the association between diabetes and stroke location. The presence of diabetes mellitus was not associated with lesions in the LCSPT circuit (*P* = 1.00). The distribution of handedness among patients with left hemisphere lesions was similar between those with and without major depressive episode. Among patients with left hemisphere lesions all the 12 (100%) patients with major depressive episode were right-handed and regarding the non-depressed patients 25 (92.6%) were right-handed and 2 (7.4%) were left-handed (*P* = 1.00)

## Discussion

In this 4-month prospective study, we found an association between the post-stroke incidence of major depressive episode and lesion volume in the left LCSPT circuit. Although this neural circuit was previously described to be affected in major depressive disorder, to our knowledge ours is the first study to directly test the importance of lesions in this circuit in relation to post-stroke depression. The importance of the LCSPT circuit activity in the pathophysiology of major depressive disorder has recently been confirmed in a catecholamine depletion study (Hasler et al. 2008). The metabolism of the LCSPT circuit was increased in remitted major depressive disorder subjects in response to catecholamine depletion but decreased or remained unchanged in healthy subjects (Hasler et al. 2008). The association of post-stroke depression with lesions in neuronal circuits has previously been investigated with MRI studies (Vataja et al. 2001, 2004). These studies reported a higher proportion and/or larger volume of infarcts in the prefrontal-subcortical circuit of post-stroke depressed patients compared with those non-depressed, with differences found particularly in the frontal lobes, caudate, pallidum, and the genu of the internal capsule, with left hemisphere predominance. The prefrontal-subcortical circuit has been associated with behavioral syndromes such as executive dysfunction, irritability, disinhibition and apathy (Cummings 1993).

Our complementary analyses revealed that five of the LCSPT areas in the left hemisphere including the ventral anterior cingulate cortex (BA24), subgenual cortex (BA25), dorsal anterior cingulate cortex (BA32), amygdala (BA34), and subiculum (BA28/ BA36) were individually associated with the post-stroke incidence of major depressive episode. The ventral anterior cingulate cortex (BA24), subgenual cortex (BA25) and the dorsal anterior cingulate cortex (BA32) are located in the medial prefrontal cortex, confirming the relevance of the frontal lobe for the pathophysiology of post-stroke depression (Robinson et al. 1984a; Mayberg et al. 1988; Vataja et al. 2001, 2004). The medial prefrontal cortex exhibits abnormal activity during periods of rest in depressive subjects (Soares and Mann 1997) and participates in the proposed default system, a network that subserves the mental processes when the individual is not engaged in any specific goal-oriented task. The medial prefrontal cortex exerts modulation over visceral control structures in the hypothalamus and brainstem, which dysfunction can lead to disturbances in autonomic regulation, as well as to neuroendocrine responses associated with mood disorders (Drevets et al. 2008; Sheline et al. 2009). The medial prefrontal cortex has been reported to be involved in the process of contextual association network (Bar et al. 2007). Lesions in the medial prefrontal cortex reduce the ability to disengage the focus of attention from one task in order to move to another task. This phenomenon would explain the depressive rumination and inability of broadly association in patients with depression (Bar 2009). The medial prefrontal cortex has also been shown to play a role in the response to treatment for depression. Hypermetabolism (Mayberg et al. 1997) and hyperactivity (Pizzagalli et al. 2001) in the rostral anterior cingulate cortex and lower metabolism in the left ventral anterior cingulate (Brody et al. 1999) have been associated with better antidepressant response. In addition, changes in medial prefrontal cortex activity might be a condition for the amelioration of depression after treatment with antidepressants (Mayberg et al. 2000), with chronic high-frequency deep brain stimulation (Johansen-Berg et al. 2008), or with cognitive behavioral therapy (Goldapple et al. 2004). It is important to note that the ventral anterior cingulate cortex (BA24), the subgenual cortex (BA25), the dorsal anterior cingulate cortex (BA32), the amygdala (BA34), and the subiculum (BA28/BA36) are also part of the medial prefrontal network and the BA34 is also present in the orbital prefrontal network. Consequently, although these networks did not present an association with post-stroke major depressive episode, lesions in the above mentioned structures may also be relevant for post-stroke major depressive episode by disrupting the medial prefrontal and the orbital prefrontal network.

The two other regions of the left LCSPT circuit in which lesion volume was associated with post-stroke incidence of major depressive episode, the amygdala and the subiculum, have also been reported to play an important role in post-stroke depression and major depressive disorder. Lesions in the amygdala have previously been associated with post-stroke depression (Vataja et al. 2001, 2004). In addition, studies of primary depression have indicated that amygdala hyperactivation (Peluso et al. 2009) is associated with depressive state, as well as that amygdala activity decreases after antidepressant treatment/symptom remission (Fu et al. 2004). Furthermore, functional coupling among the neural pathways of amygdala connections in the fronto-striato-thalamic circuits has been shown to increase after treatment with antidepressants (Chen et al. 2008). It has also been suggested that the subiculum plays a role in the pathophysiology of depression and hippocampal neuroplasticity (Bessa et al. 2009), albeit this is less extensively established than it is for the amygdala. Taking into account the relevance of the medial prefrontal cortex, subiculum, and amygdala, the consequences of stroke lesions in the LCSPT circuit, in terms of the prognosis and treatment of post-stroke depression, merit further investigation.

Our results also provide evidence that stroke lateralization is relevant for post-stroke depression. Despite some controversy (Singh et al. 1998; Carson et al. 2000; Bhogal et al. 2004; Yu et al. 2004; Hackett and Anderson 2005), major depression has been reported to be associated with left lesions in the first months after stroke (Robinson et al. 1984a; Astrom et al. 1993; Herrmann et al. 1995; Beblo et al. 1999; Vataja et al. 2001, 2004). In our study, major depressive episode was associated with the volume of lesions in the LCSPT circuit of the left hemisphere but not with the volume of lesions in neural circuits in the right hemisphere. Other studies using MRI have also supported that left hemisphere location of stroke is relevant to the occurrence and severity of depression after three to four months after stroke (Vataja et al. 2001, 2004), and a study with PET found that the ratio of ipsilateral to contralateral binding specific to S2 was correlated with severity of depressive symptoms one year after stroke in patients with left-hemisphere strokes but not those with right-hemisphere strokes (Mayberg et al. 1988). Of note, the relevance of left side location of stroke may not be generalized for all conditions of post-stroke depression. For example, a literature review suggested that left side stroke lesions contribute to the development of post-stroke depression among inpatients, in contrast to community patients, where right side lesions were associated with post-stroke depression (Bhogal et al. 2004). Similarly, left side location was relevant for the development of depression during the acute but not chronic phase after stroke. In agreement with this review, we sampled inpatients and investigated the incidence of post-stroke depression in a period of 4 months (mean 91.6 ± 5.4; 83–108 days) after stroke.

Certain limitations of our study should be considered. First, we excluded patients with hemorrhagic or infratentorial stroke, as well as those with a history of stroke or major depressive disorder. Consequently, it might not be possible to generalize our results to such patients. Second, our stroke patients were recruited from a public teaching hospital and were younger than those evaluated in studies conducted in developed countries (Conforto et al. 2008). The mean age of our sample was 51 ± 13.8 years, whereas that reported for other samples has ranged from approximately 57 to approximately 73 years (Robinson et al. 1984a; Astrom et al. 1993; Vataja et al. 2001). Third, although we investigated a highly selected sample, we did not evaluate in our study some factors that may influence the analysis including use of psychotropic medication, family history of psychiatric illness; and post-stroke social support (Robinson et al. 1984a; Astrom et al. 1993; Vataja et al. 2001, 2004). Fourth, we investigated cerebral structures using the Brodmann map (Brodmann 1909), which does not allow the investigation of white matter lesions and lesions of subcortical neuroanatomical structures. Of note, in a previous study (Vataja et al. 2004) stroke location in the pallidum was the only independent correlate of post-stroke depression in a logistic regression analysis. However, even though we did not evaluate subcortical structures in the current study, the association of post-stroke major depressive episode with lesions in the amygdala and the hippocampal subiculum, both cardinal limbic structures, reinforces the relevance of disruption of the LCSPT for the occurrence of major depressive episode after stroke. In addition, using the Brodmann map does permit a reliable comparison of cortical areas among different studies (Ongur et al. 2003). Fifth, although the MRIs were acquired in general within 2 weeks after stroke there was a large range (1–43 days) in the time interval of the MRI acquisitions. However, no time interval differences were found among the four groups of patients: left hemisphere stroke/depressed, left hemisphere stroke/non-depressed, right hemisphere stroke/depressed and right hemisphere stroke/non-depressed (*P* = 0.861). Sixth, we did not attempt to determine whether post-stroke depression was associated with other possible risk factors such as cortical and subcortical atrophy, and silent infarcts (Astrom et al. 1993; Vataja et al. 2001). It should be borne in mind that vascular risk factors such as sedentary lifestyle, smoking and the use of certain medications, none of which were included in our analysis, have also been associated with an increased risk of depression (Lustman and Clouse 2005; Iosifescu et al. 2006, 2007). Finally, any interpretation of our findings should take into consideration the fact that our analyses and results were limited to the risk of depression only during the first four months after stroke.

In conclusion, the volume of lesions affecting the LCSPT circuit in the left hemisphere, especially the ventral and dorsal anterior cingulate cortex, subgenual cortex, amygdala and subiculum, were found to be associated with major depressive episode incidence within the 4-month period following a first-ever ischemic stroke. Our findings suggest a neurobiological basis and a pathophysiological explanation for post-stroke depression.

## Statement of Interest

Dr Terroni received support from Coordenacao de Aperfeicoamento de Pessoal de Nivel Superior (CAPES) Foundation and Conselho Nacional de Desenvolvimento Cientifico e Tecnologico (CNPq). Dr Iosifescu has received research support from Aspect Medical Systems, Forest Laboratories and Janssen Pharmaceutica; he has received honoraria from Eli Lilly & Co., Forest Laboratories, Pfizer, Inc, and Reed Medical Education. The other authors report no biomedical financial interests or potential conflicts of interest.
